# All*-trans* retinoic acid enhances the cytotoxic effect of decitabine on myelodysplastic syndromes and acute myeloid leukaemia by activating the RARα-Nrf2 complex

**DOI:** 10.1038/s41416-022-02074-0

**Published:** 2022-12-08

**Authors:** Lu Wang, Qi Zhang, Li Ye, Xingnong Ye, Wenli Yang, Hua Zhang, Xinping Zhou, Yanling Ren, Liya Ma, Xiang Zhang, Chen Mei, Gaixiang Xu, Kongfei Li, Yingwan Luo, Lingxu Jiang, Peipei Lin, Shuanghong Zhu, Wei Lang, Yuxia Wang, Chuying Shen, Yueyuan Han, Xiaozhen Liu, Haiyang Yang, Chenxi Lu, Jie Sun, Jie Jin, Hongyan Tong

**Affiliations:** 1 Department of Hematology, The First Affiliated Hospital, Zhejiang University School of Medicine, 310003 Hangzhou, Zhejiang China; 2 Myelodysplastic Syndromes Diagnosis and Therapy Center, The First Affiliated Hospital, Zhejiang University School of Medicine, 310003 Hangzhou, Zhejiang China; 3 Key Laboratory of Hematologic Malignancies, Diagnosis and Treatment, 310003 Hangzhou, Zhejiang China; 4 Department of Radiotherapy, Taizhou Central Hospital (Taizhou University Hospital), 318000 Taizhou, Zhejiang China; 5 Cancer Center, Zhejiang University, 310058 Hangzhou, Zhejiang China

**Keywords:** Myelodysplastic syndrome, Cancer therapeutic resistance

## Abstract

**Background:**

Decitabine (DAC) is used as the first-line therapy in patients with higher-risk myelodysplastic syndromes (HR-MDS) and elderly acute myeloid leukaemia (AML) patients unsuitable for intensive chemotherapy. However, the clinical outcomes of patients treated with DAC as a monotherapy are far from satisfactory. Adding all-*trans* retinoic acid (ATRA) to DAC reportedly benefitted MDS and elderly AML patients. However, the underlying mechanisms remain unclear and need further explorations from laboratory experiments.

**Methods:**

We used MDS and AML cell lines and primary cells to evaluate the combined effects of DAC and ATRA as well as the underlying mechanisms. We used the MOLM-13-luciferase murine xenograft model to verify the enhanced cytotoxic effect of the drug combination.

**Results:**

The combination treatment reduced the viability of MDS/AML cells in vitro, delayed leukaemia progress, and extended survival in murine xenograft models compared to non- and mono-drug treated models. DAC application as a single agent induced Nrf2 activation and downstream antioxidative response, and restrained reactive oxygen species (ROS) generation, thus leading to DAC resistance. The addition of ATRA blocked Nrf2 activation by activating the RARα-Nrf2 complex, leading to ROS accumulation and ROS-dependent cytotoxicity.

**Conclusions:**

These results demonstrate that combining DAC and ATRA has potential for the clinical treatment of HR-MDS/AML and merits further exploration.

## Background

Myelodysplastic syndromes (MDS) are heterogeneous clonal haematopoietic disorders characterised by ineffective haematopoiesis, progressive cytopenia, and the risk of progression to acute myeloid leukaemia (AML) [1]. MDS patients can be categorised into lower-risk (LR) and higher-risk (HR) subgroups according to the International Prognostic Scoring System (IPSS) [2] and revised IPSS (IPSS-R) [3]. Aberrant DNA methylation is prevalent, and plays a pivotal role in the pathogenesis of MDS and transformation into AML [4, 5]. In HR-MDS, hypomethylating agents (HMA) are widely used as the first-line treatment to decrease the malignant clone and prolong survival. HMA are also used in elderly AML patients ineligible for intensive induction chemotherapy. However, the clinical outcomes of HR-MDS patients treated with HMA as monotherapy were unsatisfactory. The response rate of complete remission + partial remission (CR + PR) was 19–29% [6, 7], and that of CR + PR + marrow complete remission (mCR) + haematological improvement (HI) was 47.6–61% [8, 9], with an overall survival (OS) time of 17.7–22 months [10–12]. For older patients with AML (aged ≥ 60 years) treated with HMA alone, the response rate of CR + PR + CRi (morphologic CR with incomplete blood count recovery) was ~15–30%, with a median OS time of 7.7–10.4 months [13, 14]. Therefore, combination strategies were explored with the goal of achieving higher response rates and longer survival in HR-MDS and elderly AML patients. Several target agents, including BCL-2 inhibitor (venetoclax), CD47 monoclonal antibody (magrolimab), TP53 mutation modulator (APR-246), and IDH1/2 inhibitor (ivosidenib, enasidenib), used in combination with HMA (the “HMA + X” treatment strategy), are currently being investigated in various clinical trials and laboratory studies [15–19].

All*-trans* retinoic acid (ATRA) is a bioactive metabolite of vitamin A with pivotal roles in cell differentiation, proliferation, apoptosis, and embryonic development [20]. ATRA has wide applications in acute promyelocytic leukaemia (APL) patients; it targets PML/RARα infusion protein, provokes terminal differentiation of promyelocytes [21, 22], and transforms APL from a highly fatal disease to highly curable disease [20]. However, the application of ATRA in non-APL AML remains controversial.

Burnett et al. [23] reported no benefit of adding ATRA to the DAT regimen (daunorubicin, thioguanine, and Ara-C at standard or high doses) in newly diagnosed young AML and HR-MDS patients. Also, no patient subgroup achieved longer survival after the addition of ATRA [23]. Schlenk et al. reported that the addition of ATRA to ICE (idarubicin, cytarabine, and etoposide) improved the complete response rate (CRR), event-free survival (EFS), and OS in elderly patients with AML [24]. Also, clinical investigations revealed that co-administration of the hypomethylation agents decitabine (DAC) and ATRA benefitted MDS and elderly AML patients unsutiable for intensive chemotherapy [25, 26]. Moreover, DAC and ATRA combination treatment exerted a synergistic anti-leukaemia effect on AML cell lines [25, 27–29]. However, the mechanisms underlying the synergistic effect remain unclear. Therefore, we explored the effects of the combination approach of DAC and ATRA on MDS and AML cells, and the underlying molecular mechanism.

Treatment of MDS-L and MOLM-13 cells using DAC as a single agent led to nuclear factor erythroid-2 related factor 2-antioxidant response element (Nrf2-ARE) pathway activation. The Nrf2-ARE pathway, which is the primary regulator of cellular redox equilibrium, reportedly mediates resistance to chemotherapy, demethylation therapy, and target therapy in various tumours [30–33]. Previously, we showed that high expression of Nrf2 confers resistance to chemotherapy in MDS [34]. Activation of the canonical Nrf2-ARE pathway is induced by the generation and accumulation of reactive oxygen species (ROS) [35]. Once activated, the Nrf2-ARE signalling pathway restrains cellular ROS accumulation, reversing ROS-dependent cytotoxicity. Nguyen et al. reported that a relatively high dose (10 μM) of DAC promoted Nrf2 activation via mitochondrial ROS (mitoROS) induction, triggering DAC resistance in AML cells [36]. However, after MDS patients were treated with DAC at a dose of 20 mg/m^2^ for 5 consecutive days, the immediate DAC concentration in the serum was 0.76 (0.37–1.36) μM [37]. Our study showed that treatment of MDS-L and MOLM-13 cells with DAC at relatively low doses (0.8 and 1.5 μM) also led to activation of the Nrf2-ARE pathway through mitoROS accumulation, which induced transcriptional activation of the downstream antioxidant genes NQO1, DUSP1, GPX2, and FTH, thus conferring resistance to DAC. Therefore, a combination therapy that antagonises the Nrf2-ARE pathway activation is needed for an enhanced cytotoxic effect on MDS and AML.

We demonstrated that combined DAC and ATRA treatment induced apoptosis in MDS and AML cells by activating caspase proteins. Next, we determined that ATRA induced RARα activation and blocked the Nrf2-ARE pathway through the RARα-Nrf2 complex in the nucleus, thereby decreasing transcriptional activation of antioxidant genes downstream of Nrf2. We further confirmed that combined treatment decreased the tumour burden and prolonged survival in an AML murine xenograft model.

## Methods

The methods are described in more detail in the Supplementary Information.

### Cell lines and primary cells

The MDS cell line (MDS-L) was kindly gifted by Professor Guido Marcucci (City of Hope National Medical Center, Duarte, CA, USA), and the MDS transformed AML cell line MOLM-13 was from our laboratory stock and validated by short tandem repeat (STR) profiling (Supplementary Fig. 1). The bone marrow samples were taken from MDS and AML patients with at initial diagnosis at the Department of Hematology, the First Affiliated Hospital, Zhejiang University School of Medicine. The baseline data of six patients are listed in Supplementary Table 1. Informed consent was obtained from all patients. The study protocol for sample collection and clinical information was approved by the Clinical Research Ethics Committee of the First Affiliated Hospital, Zhejiang University School of Medicine. Bone marrow mononuclear cells (BMMC) were separated by density gradient centrifugation using Ficoll-Hypaque solution (TBD Science, Tianjin, China). MOLM-13 and primary cells were cultured in RPMI 1640 medium supplemented with 10% foetal bovine serum (FBS) (Vistech, Wellington, New Zealand), and the MDS-L was cultured in the indicated medium with additional IL-3 (10 ng/ml). The cell lines and primary cells were cultured at 37 °C in a humidified incubator containing 5% CO_2_. No mycoplasma contamination was observed in the cell lines mentioned above.

### Knockdown of RARA in the cell lines

The *RARA* small hairpin RNA (shRNA) (Supplementary Table 2) was subcloned via a BamHI-EcoRI restriction digest into a psi-LVRU6GP vector (GeneCopoeia, Rockville, MD, USA). The psi-LVRU6GP-scramble (GeneCopoeia) was used as a control. We used the Calcium Phosphate Cell Transfection Kit (Beyotime Biotechnology, Shanghai, China) to co-transfect recombined lentiviral vectors with the psPAX2 and pMD2 VSV-G packaging vectors in HEK293T cells. The culture supernatants were collected after 48 h and transduced onto the MDS-L and MOLM-13 cells. Infected cells were selected with puromycin (0.5–1 µg/ml) for 48 h to make stable cell lines. The expression of the target gene was quantified by quantitative polymerase chain reaction (qPCR) and western blotting.

### Co-immunoprecipitation and western blotting

We used Invent Cytoplasmic and Nuclear Extraction Kits (Invent Biotechnology) for the co-immunoprecipitation (Co-IP) experiments. The nuclear protein supernatants were collected by centrifugation at 13,000 × *g* for 1 min. The protein lysates were incubated with rabbit IgG for 30 min, followed by constant rotation with Nrf2 antibody (#12721; Cell Signaling Technology, Danvers, MA, USA) or rabbit IgG overnight at 4 °C to form an Nrf2-antibody complex. Next, we added 20 µl of resuspended Protein A/G PLUS Agarose (Santa Cruz Biotechnology, Dallas, TX, USA) to the samples to bind to the complex at 4 °C for 2 h. The immunoprecipitate was collected by centrifugation at 1000 × *g* for 5 min at 4 °C and washed three times in lysis buffer. The sample was resuspended in 30 μl of 1× electrophoresis sample buffer (ThermoFisher, Waltham, MA, USA), and boiled for 5 min. The eluted proteins were analysed by western blotting using anti-RARα (Proteintech, Wuhan, China), anti-HDAC1 (Proteintech), and anti-Nrf2 antibodies (#12721; Cell Signaling Technology).

### In vivo murine xenograft models

All animal experiments were approved by the First Affiliated Hospital, Zhejiang University School of Medicine (Hangzhou, China) and conducted following the National Institutes of Health Guide for the Care and Use of Laboratory Animals. For the mouse model, 5-week-old female NOD-Prkdcscid IL2rgtm1/Bcgen (B-NDG) mice (Biocytogen, Wakefield, MA, USA) were maintained under specific pathogen-free conditions, housed in isolated vented cages, and handled using aseptic procedures for 1 week. Next, 1 × 10^6^ MOLM-13-Luciferase (MOLM-13-Luc) cells were injected via the tail veins to establish the cell line-derived xenograft (CDX) model. Leukaemia engraftment was assessed by intraperitoneal injection of D-luciferin (150 mg/kg), followed by bioluminescent imaging using the IVIS Lumina LT system (PerkinElmer, Waltham, MA, USA). Twenty-four mice were randomly assigned to four groups and treated with either 3.3 mg/kg DAC, 10 mg/kg ATRA, both drugs in combination at the indicated concentrations (COM), or vehicle (Ctrl). DAC was diluted in PBS to 3.3 mg/ml and stored at −20 °C. ATRA was prepared in 0.01% carboxymethyl cellulose sodium (CMC-Na) and stored at −20 °C. ATRA was administered by gastric lavage for 21 days. DAC was administered by tail-vein injection over 5 days. The mice were humanely sacrificed after observation of bowed back and paralysed limbs. Survival curves were generated using GraphPad Prism 8 (GraphPad Software Inc., San Diego, CA, USA), and differences between groups were analysed using two-tailed Student’s t-tests. The spleens were harvested for hematoxylin and eosin (HE) staining and immunohistochemistry (IHC). Human CD45 antibody (#13917; Cell Signaling Technology) was used to identify the engraftments.

### Statistical analysis

Data were visualised and analysed using GraphPad Prism 8 software and SPSS 20.0 (IBM Corp., Armonk, NY, USA). The values represent the means ± standard deviation (SD) of at least three independent experiments. Statistical analyses were performed using the unpaired Student’s *t*-test for two-group comparisons. We used analysis of variance (ANOVA) for comparison of more than two groups. Survival comparisons were performed using the Kaplan–Meier method and analysed using the log-rank test. *P*-values < 0.05 were considered statistically significant.

## Results

### ATRA augmented the cytotoxic effect of DAC on MDS and AML cells

**Fig. 1:**
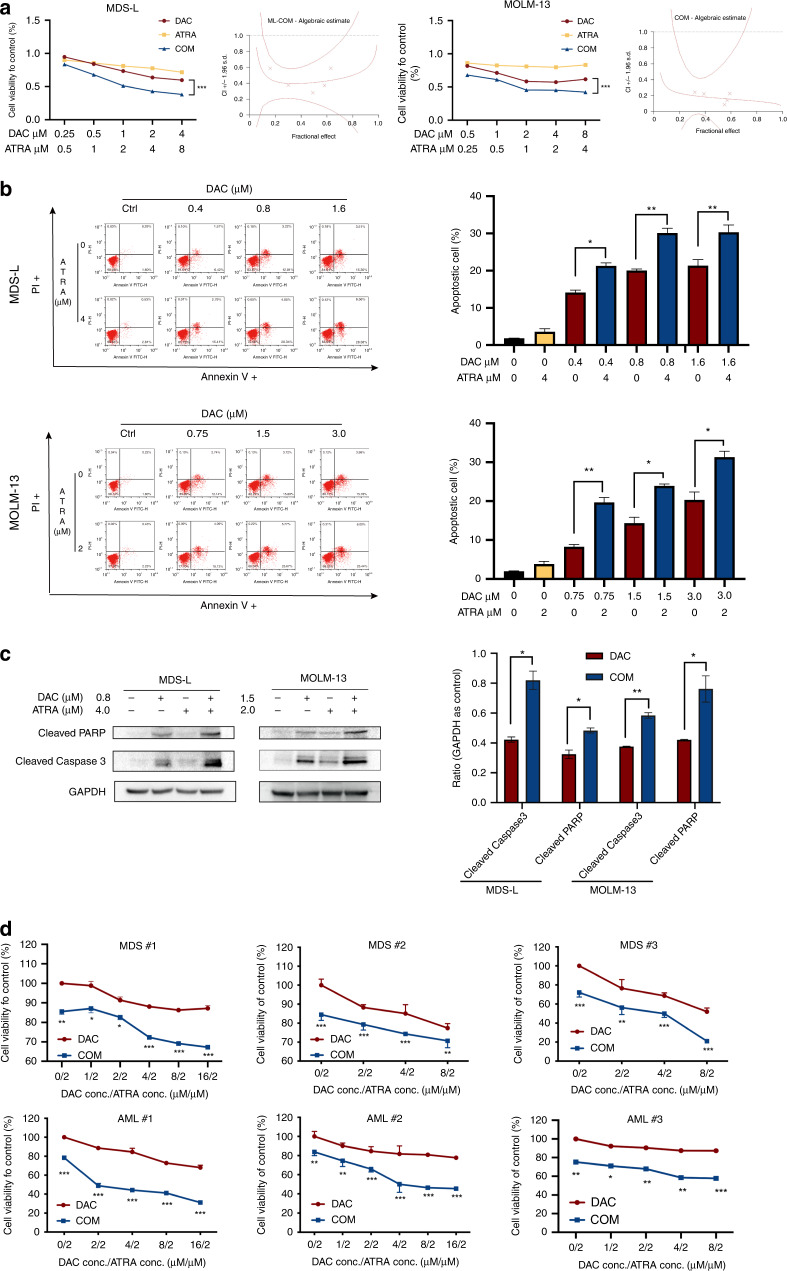
Effect of DAC and ATRA on cellular viability and apoptosis in MDS and AML cells. **a** Co-treatment with DAC and ATRA inhibited the cellular viability of MDS-L and MOLM-13 cells. The cells were measured by CellTiter-Lumi™ Assay after treatment with DAC and/or ATRA at the indicated concentrations for 48 h. **b** Combined treatment with DAC and ATRA enhanced the apoptosis of MDS-L and MOLM-13 cells. The cells were treated with DAC and/or ATRA at the indicated concentrations for 48 h and co-stained with annexin V and PI, after which apoptosis was measured by flow cytometry. The results are presented as bar graphs (*n* = 3). **c** The expression of cleaved caspase-3 and cleaved PARP increased significantly after treatment with a combined strategy compared to the single drug treatment. The expression of cleaved caspase-3 and cleaved PARP was determined by western blotting and quantified by ImageJ (NIH, Bethesda, MD, USA). GAPDH served as a loading control. **d** Co-treatment with DAC and ATRA significantly inhibited cell viability in primary blast cells of MDS and AML patients as measured by a CellTiter-Lumi™ Assay. The data are presented as the mean ± SD from three independent experiments. * *P* < 0.05, ** *P* < 0.01, *** *P* < 0.001.

We treated MDS-L and MOLM-13 cell lines with DAC at doses consistent with the serum concentrations of patients under standard treatments (DAC at 20 mg/m^2^ for 5 days). Treatment with DAC or ARTA alone had limited effects on the viability and apoptosis of MDS-L and MOLM-13 cell lines. However, exposure to both agents decreased the viability of MDS-L and MOLM-13 cells, as measured by the CellTiter-Lumi™ Assay. The Chou-Talalay method was used to calculate the combination index (CI) of the two drugs, and yielded a value <1 (indicating a synergistic effect; Fig. 1a). We also observed enhanced apoptosis (Fig. 1b) in the co-treatment group, as evidenced by increased annexin V/PI staining (*P* < 0.05) and upregulated cleaved caspase-3 and cleaved PARP (Fig. 1c). The cell cycle distribution showed no significant difference between the mono-drug and combination groups (Supplementary Fig. 2). Therefore, the decreased cell viability was mainly attributed to the pro-apoptosis effect. We also examined the cell viability of primary cells from newly diagnosed MDS and AML patients after exposure to a single agent and both in combination. The combined treatment with DAC and ATRA reduced cell viability compared to the mono-drug treatments (Fig. 1d).

### DAC and ATRA increased the cellular cytotoxicity by elevating mitoROS levels

**Fig. 2:**
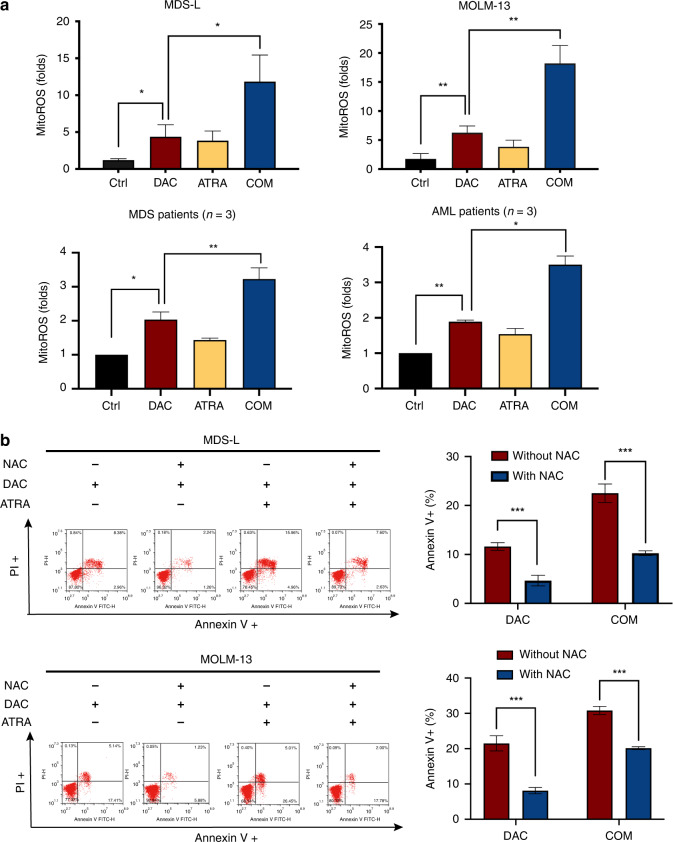
MitoROS elevation induced cellular apoptosis in MDS and AML. **a** Combination treatment with DAC and ATRA markedly elevated the mitoROS level compared with DAC alone (*P* < 0.05). MDS-L and MOLM-13 cells were treated with DAC (0.8 and 1.5 μM, respectively) or ATRA (4 and 2 μM, respectively), or both drugs in combination. Primary cells of MDS and AML were treated with DAC (1 μM) or ATRA (1 μM) alone, or both drugs in combination. After exposure for 48 h, mitoROS levels were measured by MitoSOX Red staining. **b** Pre-treatment with NAC of MDS and AML cells reversed the apoptosis induced by DAC alone and the combined DAC and ATRA treatment (*P* < 0.05). MDS-L and MOLM-13 cells were pre-treated with 2.5 mM NAC for 2 h and subsequently treated with DAC, alone or combined with ATRA, at the indicated concentrations for 48 h. Apoptosis measurements are shown (*n* = 3). NAC N‐acetyl‐l‐cysteine, ROS reactive oxygen species.

A relatively high DAC dose (10 μM) exerts a cytotoxic effect via mitoROS induction. We investigated whether the cytotoxicity induced by DAC at lower doses (0.8 and 1.5 μM on cell lines and 1.0 μM on primary cells), as a single agent and combined with ATRA, was related to mitoROS. DAC alone induced mitoROS production in MDS-L, MOLM-13 and primary cells. DAC and ATRA combined augmented mitoROS generation (Fig. 2a). Pre-treatment with the free radical scavenger N-acetyl-L-cysteine (NAC) reversed the apoptotic effects of DAC, alone and in combination with ATRA (Fig. 2b), implicating the mitoROS elevation induced by ATRA in the greater cytotoxicity of the combined treatment.

### ATRA blocked the Nrf2-ARE activation induced by a low dose of DAC

**Fig. 3:**
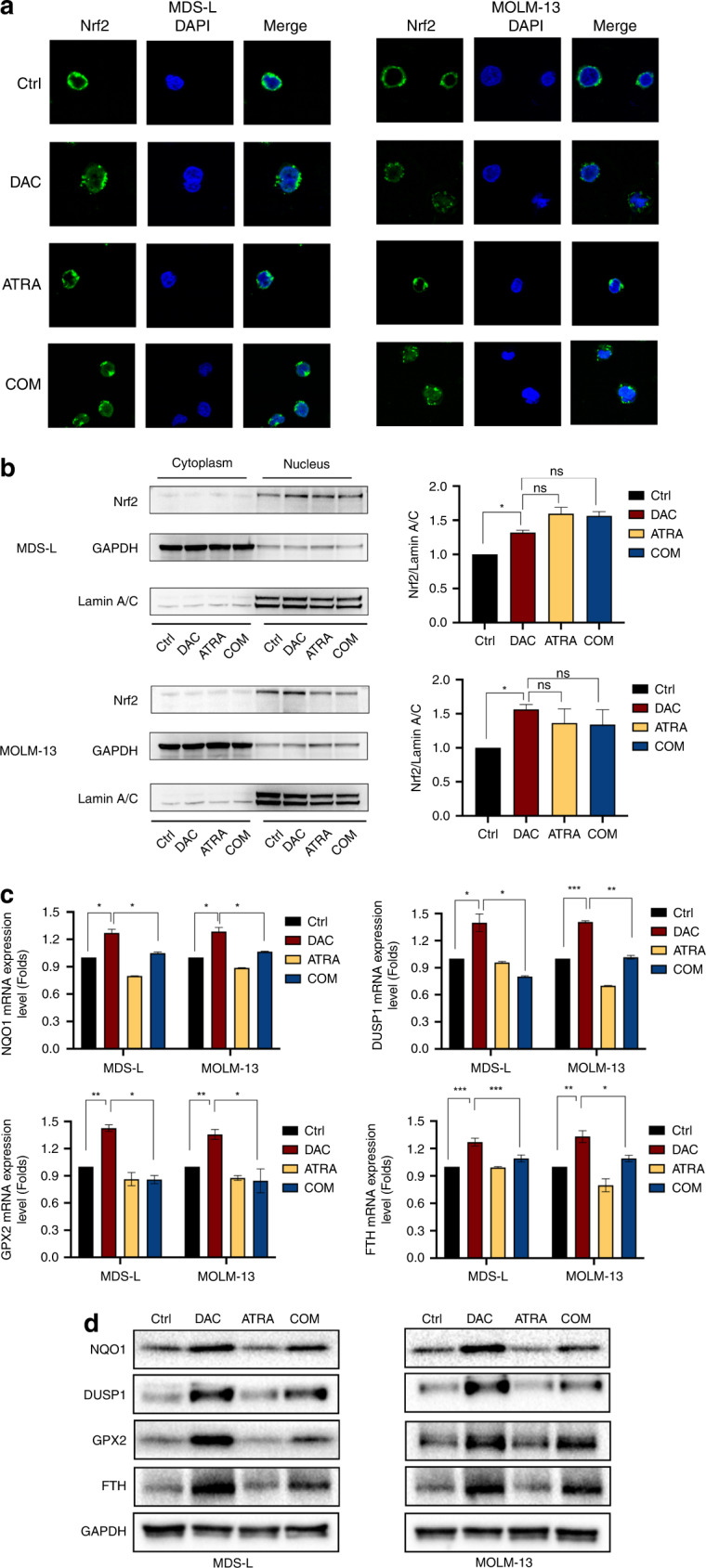
Combined treatment with DAC and ATRA restrained DAC-mediated Nrf2 activation. MDS-L and MOLM-13 cells were treated with DAC (0.8 and 1.5 μM, respectively), ATRA (4 and 2 μM, respectively), or both drugs in combination for 24 h. Immunofluorescence (**a**) and immunoblotting analysis (**b**) showed that DAC treatment induced Nrf2 translocation into the nucleus. Co-treatment with DAC and ATRA did not block the translocation triggered by DAC. Compared with the Ctrl, treatment with DAC alone significantly increased the mRNA (**c**) and protein (**d**) expression levels of NQO1, DUSP1, GPX2, and FTH in MDS-L and MOLM-13 cells. At the same time, the combination of DAC and ATRA markedly reversed the upregulated expression caused by DAC.

The Nrf2-ARE pathway is the primary regulator of cellular redox equilibrium. To investigate the correlation between Nrf2 activation and mitoROS, we performed immunofluorescence analyses of MDS-L and MOLM-13 cells. DAC treatment translocated Nrf2 from the cytoplasm to the nucleus, indicating that Nrf2 was activated by DAC (Fig. 3a). Using cellular fraction immunoblotting, we found that DAC activated the Nrf2-ARE pathway, thereby triggering Nrf2 relocation to the nucleus (Fig. 3b). Consistent with Nrf2 activation, we observed increased mRNA and protein expression levels of the Nrf2-dependent NQO1, DUSP1, GPX2, and FTH in DAC-treated cells (Fig. 3c, d). Moreover, co-treatment with DAC and ATRA markedly inhibited the expression of the Nrf2-dependent genes NQO1, DUSP1, GPX2, and FTH (Fig. 3c, d) but did not reverse Nrf2 translocation (Fig. 3a, b), suggesting that the antagonism of ATRA to Nrf2 occurred in the nucleus in a post-translocation manner. Therefore, we hypothesised that repressing Nrf2-ARE activation could stimulate the mitoROS‐mediated cytotoxic effect of DAC.

### Knockdown of RARα counteracted ATRA-induced apoptosis

**Fig. 4:**
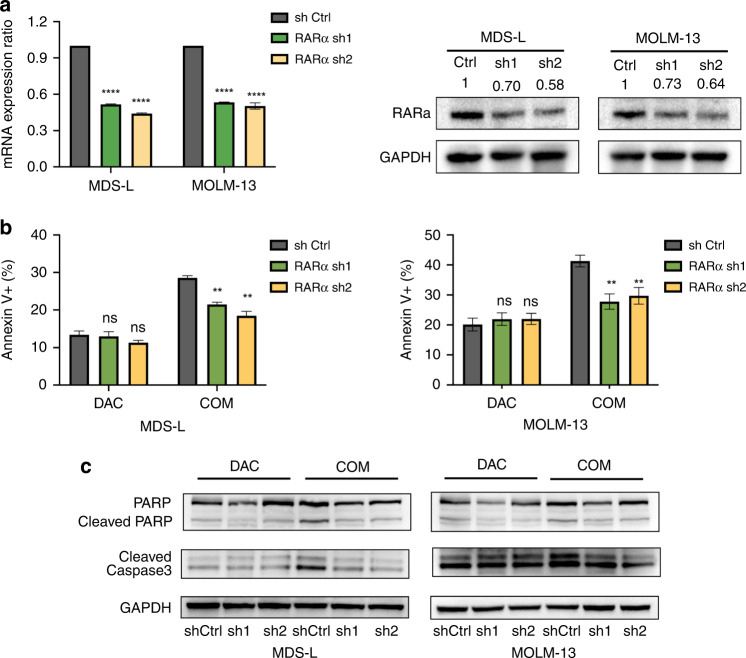
Effect of RARα KD on cellular apoptosis in MDS-L and MOLM-13 cells. **a** Transcriptional levels of RARα in sh1 and sh2 cells were significantly reduced compared with the shCtrl. Immunoblots with anti‐RARα and anti‐GAPDH antibodies showed that RARα protein expression was markedly decreased in sh1 and sh2 cells. Flow cytometry staining with annexin V-APC (**b**) and western blotting results (**c**) showed that RARα KD reversed the apoptosis induced by the combined treatment, but not that induced by DAC alone. MDS-L and MOLM-13 cells were treated with DAC (0.8 and 1.5 μM, respectively) or DAC and ATRA (4 and 2 μM, respectively) for 48 h.

ATRA exerts pleiotropic effects by activating its RARα ligand. We examined the effect of RARα knockdown (KD) by shRNA on apoptosis to determine the role of RARα in synergistic apoptosis. Real-time PCR and western blotting (Fig. 4a) showed that the shRNAs sh1 and sh2 markedly decreased RARα expression compared with the shCtrl. Annexin V/PI staining showed that RARα KD reversed the cytotoxicity of the combination group compared with the shCtrl. However, there was no significant difference in the apoptosis rate between RARα KD and shCtrl cells when treated with DAC alone (Fig. 4b, c). These results indicated that ATRA restrained the Nrf2-ARE signalling response by activating RARα.

### Combined treatment activated the RARα-Nrf2 functional complex in the nucleus

**Fig. 5:**
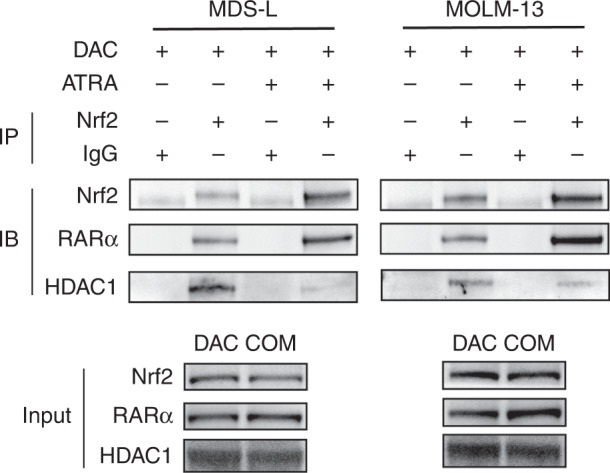
RARα-Nrf2 complex activation after combined treatment with DAC and ATRA. Immunoprecipitation and immunoblotting indicated the formation of a RARα-Nrf2 complex in MDS-L and MOLM-13 cells treated with DAC, as a single agent or combined with ATRA. The expression level of HDAC1 binding to the complex was reduced after the combination treatment.

We performed immunoprecipitation experiments to investigate whether activated RARα antagonised Nrf2 function through physical interactions. After treatment with DAC, alone or in combination with ATRA for 24 h, we used the Nrf2 antibody to immunoprecipitate Nrf2 in MDS-L and MOLM-13 cells. Immunoblots of the precipitate revealed the presence of RARα and HDAC1 (Fig. 5), suggesting that Nrf2, RARα, and HDAC1 formed a complex. Nrf2 coprecipitated with RARα in the presence of DAC, either as a single agent or with the addition of ATRA. However, the level of HDAC1 bound to the RARα-Nrf2 complex decreased after co-treatment with DAC and ATRA, suggesting that the RARα-Nrf2 complex was activated and counteracted the activation of Nrf2-ARE signalling.

### Combined DAC and ATRA treatment showed antileukemic activity in vivo

**Fig. 6:**
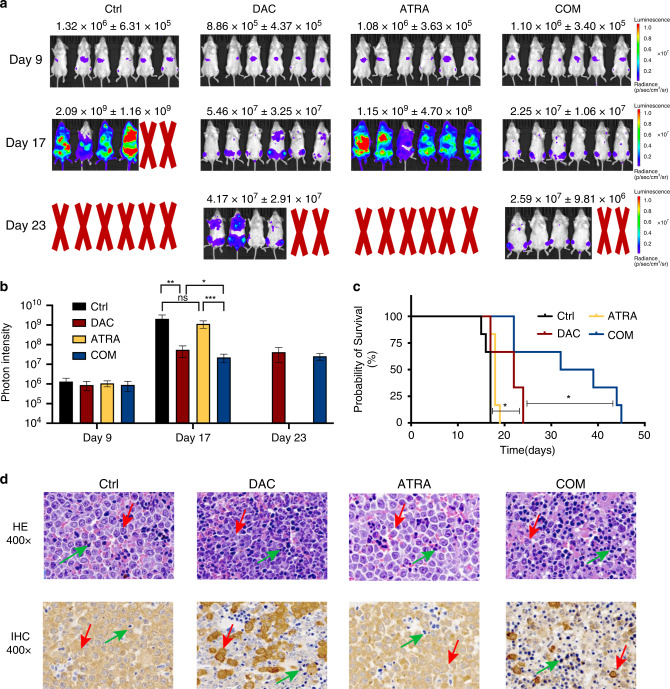
Combined treatment reduced the tumour burden and prolonged survival in a murine xenograft model. **a** Compared with the Ctrl and mono-drug group, the combined treatment with DAC and ATRA significantly reduced the tumour burden in a murine model. **b** The leukaemia burden was lowest in the COM group on Day 17, as indicated by the photon intensity (*P* < 0.05). **c** Co-treatment with DAC and ATRA significantly improved the survival time in the murine model (*P* < 0.05). **d** HE and IHC staining of the murine spleens showed that the combined DAC and ATRA regimen reduced malignant cell infiltration in vivo. Red arrow, MOLM-13-Luc cells; green arrow, murine spleen cells.

We evaluated the effect of combining DAC and ATRA on leukaemia burden and survival in a murine xenograft model. The model was established by intravenous injection with luciferase-labelled MOLM-13 cells via the tail vein. The mice were treated with vehicle, DAC (3.3 mg/kg/day for 5 days), ATRA (10 mg/kg/day for 21 days), or both drugs in combination. We calculated the DAC dose for the murine model based on the typical DAC dose applied to patients, while the dose of ATRA was consistent with previous articles [38, 39]. On day 17, DAC administration reduced the leukaemia burden, while ATRA had no apparent effect. Compared with the single-agent treatments, the combined treatment remarkably reduced the tumour burden, manifested in a significant reduction in bioluminescence (Fig. 6a). The leukaemia burden was lowest in the COM group, as indicated by the photon intensity (Fig. 6b, *P* < 0.05).

Although the mice treated with DAC alone survived longer than those who received the vehicle or ATRA, the combined DAC and ATRA regimen led to significantly longer survival than DAC only (median survival time of 32 days for the combination group vs. 22 days for DAC monotherapy group, *P* < 0.05, Fig. 6c). Hematoxylin–Eosin (HE) and immunohistochemistry (IHC) staining showed that the DAC and ATRA combination treatment markedly reduced the number of leukaemia cells infiltrating the spleen (Fig. 6d). This was consistent with the photon intensity results showing that DAC and ATRA synergistically decreased the leukaemia burden. Therefore, we showed that the DAC and ATRA combination is a promising treatment regimen for HR-MDS and AML patients.

## Discussion

**Fig. 7:**
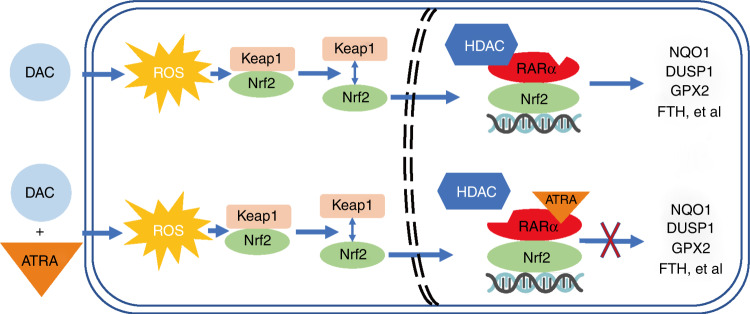
A proposed model of the RARα-Nrf2 complex in MDS and AML cells exposed to DAC, alone or combined with ATRA. Treatment with DAC alone induced ROS generation and activated the Nrf2-ARE antioxidant response. Co-treatment with DAC and ATRA reduced the level of HDAC1 binding to the RARα-Nrf2 complex, prevented the activation of Nrf2 by DAC alone, and blocked transcriptional activation of Nrf2 downstream antioxidant genes.

Our results show that DAC and ATRA exert synergistic cytotoxic effects in MDS and AML cells. Treatment with DAC alone activated the antioxidant Nrf2-ARE pathway in MDS and AML cells. However, when combined with ATRA, it activated the RARα-Nrf2 complex and blocked Nrf2 activation, leading to ROS accumulation and ROS-dependent anti-tumour effects. Based on these data and those of previous studies, we propose a mechanism underlying DAC resistance mediated by the RARα-Nrf2 complex (Fig. 7).

Previous studies suggests that the combination of DAC and ATRA synergistically induces apoptosis, growth inhibition, and differentiation of AML cells by inducing the expression of anti-tumour genes [28] and modulating the miR-34a/MYCN axis in vitro [25]. Consistent with previous reports, the DAC and ATRA combination treatment exerted a synergistic anti-tumour effect on both cell lines and primary samples of MDS and AML patients. The doses of ATRA used to treat the MDS-L and MOLM-13 cell line models (4 and 2 μM, respectively) were higher than the ATRA concentrations usually used to treat MDS/AML cell lines [38, 40–43] (1 or <1 μM). However, in this study, a low ATRA concentration of <4 μM (for MDS-L) or <2 μM (for MOLM-13) triggered no pro-apoptotic and anti-growth effects; the sensitivity to ATRA may vary across cell lines. Moreover, in a previous study of ATRA pharmacokinetics, 11 healthy volunteers were intravenously administered liposomal (L)-ATRA (90 mg/m^2^ every other day over 15 days) and the maximum plasma ATRA concentration was 8146 ng/ml (27.1 μM). L-ATRA maintained a stable plasma concentration with moderate adverse effects. The doses that we used to treat the MDS-L and MOLM-13 cell lines were far lower than that mentioned above, suggesting that our doses have low toxicity and are well-tolerated [44].

Our in vivo experiment showed that the combined treatment markedly reduced the leukaemia burden and improved survival in a MOLM-13 xenograft B-NDG model compared with DAC alone. Previously, a leukaemic murine model was administered ATRA-release pellets. Once implanted subcutaneously, the pellets were designed to release ATRA over 21 days at an average dose of 0.23, 0.5, or 1.2 mg/day [45]. In our study, mice were treated with ATRA by gastric lavage, at a dose of 10 mg/kg for 21 days. As the average mouse weighs ~20 g, the average ATRA dose of each mouse was about 0.2 mg/day, similar to the minimal dose described above.

Incubation of MDS-L and MOLM-13 cells with ATRA in the presence of DAC showed that ATRA counteracted the activation of the Nrf2-ARE antioxidant pathway, caused ROS accumulation and ROS-dependent cytotoxicity, and thereby overcame DAC resistance. Under homoeostatic conditions, Nrf2 localises in the cytoplasm and binds to its repressor Kelch-like ECH-associated protein 1 (Keap1), which targets Nrf2 for proteasomal degradation. In response to oxidant stress, Nrf2 is released from Keap1 and translocates to the nucleus [46–48]. Next, Nrf2 interacts with ARE to induce the expression of Nrf2-responsive genes [49]. However, the mechanism through which ATRA inhibits Nrf2 is unclear, and the results of previous studies of different tumours differ. Valenzuela et al. reported that ATRA prevented Nrf2 from translocating into the nucleus in APL cells, thus counteracting Nrf2-ARE activation [50]. However, Wang et al. suggested that ATRA blocked post-translocational Nrf2 activity in the MCF-7 breast cancer cell [51]. Simialr to the MCF-7 cell results, ATRA prevented transcriptional activation of cytoprotective and detoxifying genes NQO1, DUSP1, GPX2, and FTH downstream of Nrf2-ARE in the MDS-L and MOLM-13 cells, without inhibiting Nrf2 translocation to the nucleus.

ATRA interacts with retinoic acid receptors (RARs) and has pleiotropic effects [52]. RARs are ligand-dependent transcription factors that specifically regulate retinoic acid signalling by forming heterodimers with the retinoid X receptor (RXR) [53]. RAR/RXR heterodimers influence transcriptional activation by binding and recruiting co-activators [53, 54] and co-repressors [55, 56]. Co-repressors such as nuclear receptor co-repressor (NCoR) recruit large repressor complexes, including histone deacetylases (HDACs), thereby inhibiting the transcription of target genes [57–59]. RARα KD in MDS-L and MOLM-13 cells reversed the apoptosis induced by the DAC and ATRA combination treatment, but not that induced by DAC alone. A Co-IP assay confirmed the presence of RARα-Nrf2-HDAC1 after treatment with DAC, alone and in combination with ATRA. The HDAC1 expression level of the combination group was significantly lower than that of the single DAC group, suggesting that ATRA activated the RARα-Nrf2 complex in the presence of DAC. Moreover, Wang et al. reported that ATRA exposure reduced Nrf2-ARE binding [51]. Given the previous reports, we suggest that the activated RARα-Nrf2 complex carrying less HDAC1 exhibited less affinity for the ARE enhancer. Therefore, Nrf2 activation was inhibited, resulting in mitoROS induction and enhanced cytotoxicity.

This study showed that DAC and ATRA synergistically enhanced apoptosis in MDS and AML cells, in vivo and in vitro. Based on these findings, a multi-center clinical trial is currently underway at our center to explore the effect of the combination of DAC and ATRA on newly diagnosed MDS with excess blast (MDS-EB) patients (ChiCTR1800018307). Interestingly, an ongoing clinical trial (NCT02807558) sought to determine whether the selective RARα agonist SY-1425 [60] and azacytidine (AZA) could benefit AML and MDS patients. The preliminary data showed that the combination treatment benefitted newly diagnosed and relapsed/refractory AML patients [61, 62].

This study had some limitations. Firstly, we did not construct a patient-derived xenograft (PDX) model to explore the in vivo effects of the drug combination. Moreover, single doses of DAC and ATRA were applied in the murine model, resulting in a lack of comparison of the effects of different concentrations on leukaemia burden and survival. In addition, we did not perform RNA sequencing. Since we collected the primary samples from patients enroled in the ongoing clinical trial, these primary samples will be sequenced in a follow-up study. We hope that this will reveal additional molecular mechanisms meriting further exploration. Finally, the current study was limited by its preclinical nature, further randomised controlled trials of patients with MDS and AML are needed to validate the results.

In conclusion, the current study indicates that DAC and ATRA in combination synergistically induce apoptosis in MDS and AML cells, thereby showing an enhanced anti-tumour effect in vivo and in vitro. Mechanistically, as a single agent, DAC activated the antioxidant Nrf2-ARE signalling pathway in MDS and AML cells, leading to DAC resistance. ATRA blocks Nrf2 activation by activating the RARα-Nrf2 complex, and causes ROS accumulation and ROS-dependent cytotoxicity. Therefore, a regimen combining DAC and ATRA for HR-MDS and AML has clinical potential and merits further exploration.

## Supplementary information

Supplementary data

## Data Availability

All data supporting the conclusions of this study have been included within the article and the supplemental data.
