# Supplementary material for: All-trans retinoic acid enhances the cytotoxic effect of decitabine on myelodysplastic syndromes and acute myeloid leukaemia by activating the RARα-Nrf2 complex
Source: Br J Cancer. 2022 Dec 8;128(4):691–701. doi: 10.1038/s41416-022-02074-0 (PMC9938271; doi:10.1038/s41416-022-02074-0)
Supplement: Supplementary file 1 — Supplementary data [file 41416_2022_2074_MOESM1_ESM.docx]

**Supplementary Methods**

**Reagents**

Decitabine (S1200) was purchased from Selleck Chemicals (Texas, USA). Dimethyl sulfoxide (DMSO, D2650), and all-trans retinoic acid (ATRA, T1850000) were purchased from Sigma (Missouri, USA). The Nrf2 (12721), poly ADP-ribose polymerase (PARP, 9532S), caspase 3 (9962S) antibodies, and anti-rabbit and anti-mouse antibody (7074S and 7076S, respectively), which served as secondary antibodies, were obtained from Cell Signaling Technology ([Massachusetts](http://www.baidu.com/link?url=Jipxly9GwSoSz1CDTzAGTof2D4dxyYaSM0B5uHllbxIEeJyK_fiiVkp_aVC1MTHtv0z1yJVmnGga4Yn7QLc4hLRkqAVIPy5ycajaC0cL4C3), USA). The dual-specificity protein phosphatase-1 (DUSP1, 07-535) antibody was obtained from Merck company (Darmstadt, Germany), and the NAD(P)H: quinone oxidoreductase 1 (NQO1, ab28947), ferritin H (FTH, ab65080), and glutathione peroxidase 2 (GPX2, ab137413) antibodies were purchased from Abcam company (Cambridge, UK). The Nrf2 antibody (ab31163) used for immunofluorescence (IF) were bought from Abcam (Cambridge, UK). The RARα (10331-1-AP), histone deacetylase 1 (HDAC1, 10197-1-AP), and glyceraldehyde-3-phosphate dehydrogenase (GAPDH, 60004-1-Ig) were purchased from Proteintech Technology (Wuhan, China).

**Cell viability assays**

Primary cells (1×10^5^) were seeded in 96-well opaque plates containing 100μl culture medium and treated with designated doses of DAC and ATRA alone or in combination for 48h. The plate was equilibrated to room temperature for 30 minutes before adding 100μl CellTiter-Lumi™ Assay (Beyotime biotechnology, Shanghai, China) to each well. After mixing for 2 minutes on an orbital shaker, the contents were incubated at room temperature for 10 minutes, after which the luminescence was detected by a microplate reader (Varioskan Flash, Thermo Fisher Scientific, [Massachusetts](http://www.baidu.com/link?url=Jipxly9GwSoSz1CDTzAGTof2D4dxyYaSM0B5uHllbxIEeJyK_fiiVkp_aVC1MTHtv0z1yJVmnGga4Yn7QLc4hLRkqAVIPy5ycajaC0cL4C3), USA).

**Western blotting analysis**

 After different treatments, a total of 2×10^6^/ml cells per condition were washed with ice-cold phosphate-buffered saline (PBS). Mix Western and IP buffer (Beyotime biotechnology, Shanghai, China) and protease inhibitor cocktail (Thermo Fisher Scientific, [Massachusetts](http://www.baidu.com/link?url=Jipxly9GwSoSz1CDTzAGTof2D4dxyYaSM0B5uHllbxIEeJyK_fiiVkp_aVC1MTHtv0z1yJVmnGga4Yn7QLc4hLRkqAVIPy5ycajaC0cL4C3), USA) at the ratio of 100:1. Each sample was added with 100μl mixed buffer and put on ice for 30 minutes. The protein concentrations were determined using a BCA protein assay kit (Beyotime biotechnology, Shanghai, China). The protein samples were mixed with 4× loading buffer and were denatured by boiling at 100°C for 5 minutes before separation on 4-20% SDS-PAGE gels. The separated proteins were then transferred to 0.2 or 0.45μm polyvinylidene difluoride (PVDF) membranes (Millipore, [Massachusetts](http://www.baidu.com/link?url=Jipxly9GwSoSz1CDTzAGTof2D4dxyYaSM0B5uHllbxIEeJyK_fiiVkp_aVC1MTHtv0z1yJVmnGga4Yn7QLc4hLRkqAVIPy5ycajaC0cL4C3), USA). The target protein bands were visualized using an ECL kit (Thermo Fisher Scientific, [Massachusetts](http://www.baidu.com/link?url=Jipxly9GwSoSz1CDTzAGTof2D4dxyYaSM0B5uHllbxIEeJyK_fiiVkp_aVC1MTHtv0z1yJVmnGga4Yn7QLc4hLRkqAVIPy5ycajaC0cL4C3), USA) and detected using the ChemiDoc MP Imaging System (Bio-Rad, California, USA).

**Flow cytometric analysis**

For apoptosis assessment, 2×10^5^ cells were seeded per well in 24-well plates. DAC and ATRA as a single agent or in combination were added to each well at appropriate concentrations for 48 hours. After that the cells were washed with 1×PBS and resuspended with 1×binding buffer. Each sample was co-stained with Annexin-V FITC and PI using apoptosis detection kit (Mulisciences, Hangzhou, China) for 10 minutes at room temperature in the dark. For sh-RARA cells, Annexin-V APC and PI were used to detect apoptosis according to the protocol described previously. Cells were analyzed using with NovoExpress software (ACEA Biosciences, California, USA) to examine the percentage of apoptotic cells.

MitoSOX Red (Invitrogen, California, USA) was used to analyze ROS production. The cells were treated with different drugs for 48h then collected by centrifugation at 300g ×5minutes and resuspended in 5μM MitoSOX Red working solution for 10 minutes at 37℃ in the dark, after which, each sample was washed gently with warm PBS and resuspended with 100μl PBS before analyzed by flow cytometry.

**Real time reverse transcription PCR (qRT-PCR)**

Total cellular RNA was extracted using the MolPure® TRIeasy Plus Total RNA Kit (YEASEN Biotech, Shanghai, China) and was reverse transcribed into cDNA using PrimerSctipt RT agent Kit (Takara, Dalian, China) according to the manufacturer’s protocol. The Quantitative assessments of amplified cDNA was performed on an Applied Biosystems CFX96 real-time PCR system (Bio-Rad, California, USA) using the TB-Green PCR Master Mix kit (Takara, Dalian, China). The primers sequences were as follows:

*RARA* (forward:5’-GGGCAAATACACTACGAACAACA-3’;

reverse:5’- CTCCACAGTCTTAATGATGCACT-3’);

*NQO1* (forward: 5’-GAAGAGCACTGATCGTACTGGC-3’;

reverse: 5’- GGATACTGAAAGTTCGCAGGG-3’),

*DUSP1*(forward:5’-ACCACCACCGTGTTCAACTTC-3’;

reverse:5’-TGGGAGAGGTCGTAATGGGG-3’)

*GPX2*(forward: 5’-GGTAGATTTCAATACGTTCCGGG;

reverse:5’- TGACAGTTCTCCTGATGTCCAAA)

*FTH* (forward: 5’-TCCTACGTTTACCTGTCCATGT;

reverse:5’- GTTTGTGCAGTTCCAGTAGTGA)

*GAPDH* (forward:5’-GGAGCGAGATCCCTCCAAAAT-3’;

reverse: 5’- GGCTGTTGTCATACTTCTCATGG-3’).

The relative gene expression was calculated by the 2^−ΔΔCt^ method and normalized to the expression of *GAPDH*.

**Immunofluorescence microscopy**

After treatment with single agent or combination for 24 hours, the MDS-L and MOLM-13 cells were cytospun (400g for 5 minutes) onto a poly-L-lysine-coated glass. After fixation in 4% paraformaldehyde for 10 minutes and permeabilization in 0.3% Triton X-100/PBS for 15 min, 1% bovine serum albumin (BSA) was used to block the non-specific antigen on cell surface for 1 ­hour. After that, the Nrf2 primary antibody (ab31163, Cambridge, UK) at 1:500 dilution was used for incubation overnight at 4°C. Secondary antibody staining was done with Goat anti rabit IgG H&L Alexa Fluor® 488 (ab150070, Cambridge, UK) goat anti-rabbit antibodies at a 1:500 dilution. Nuclei were stained with DAPI in a concentration of 0.5μg/ml (#4083, Cell signaling technology, [Massachusetts](http://www.baidu.com/link?url=Jipxly9GwSoSz1CDTzAGTof2D4dxyYaSM0B5uHllbxIEeJyK_fiiVkp_aVC1MTHtv0z1yJVmnGga4Yn7QLc4hLRkqAVIPy5ycajaC0cL4C3), USA), which were performed after the secondary antibody incubation step. Images were captured with the Leica TSC SP8 confocal laser scanning microscope (Leica, Wetzlar, Germany) coupled to a Leica DMi8 inverted microscope (Leica, Wetzlar, Germany).

**Hematoxylin-eosin (HE) staining**

The spleen tissues were embedded in paraffin and cut into 4μm serial sections. The spleen tissue sections were dewaxed and stained with hematoxylin-eosin (HE) staining kit. Images of immunohistochemistry were obtained under a light microscope. Histological morphology was analyzed to investigate leukemic cells infiltration.

**Supplementary Figure 1**


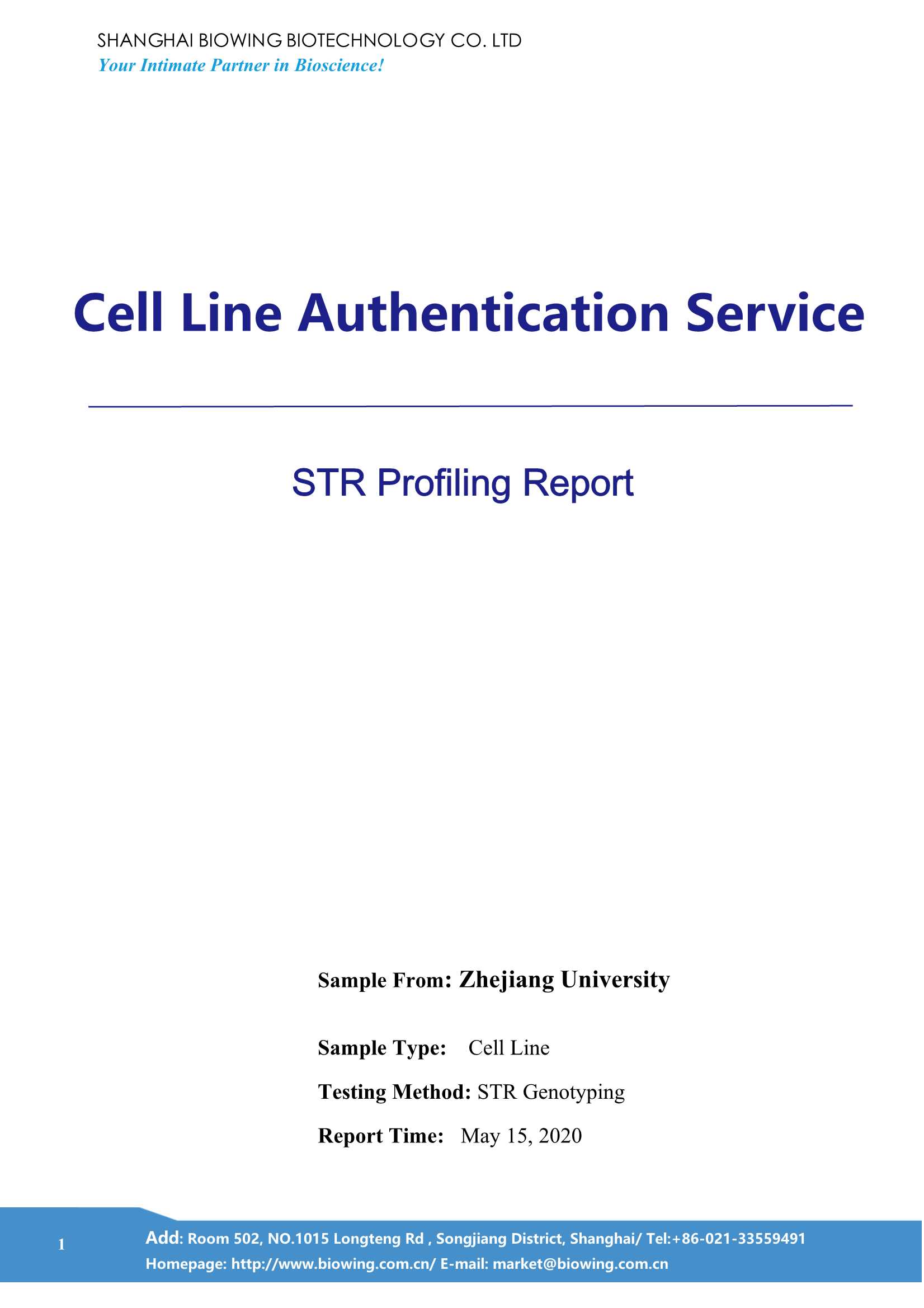


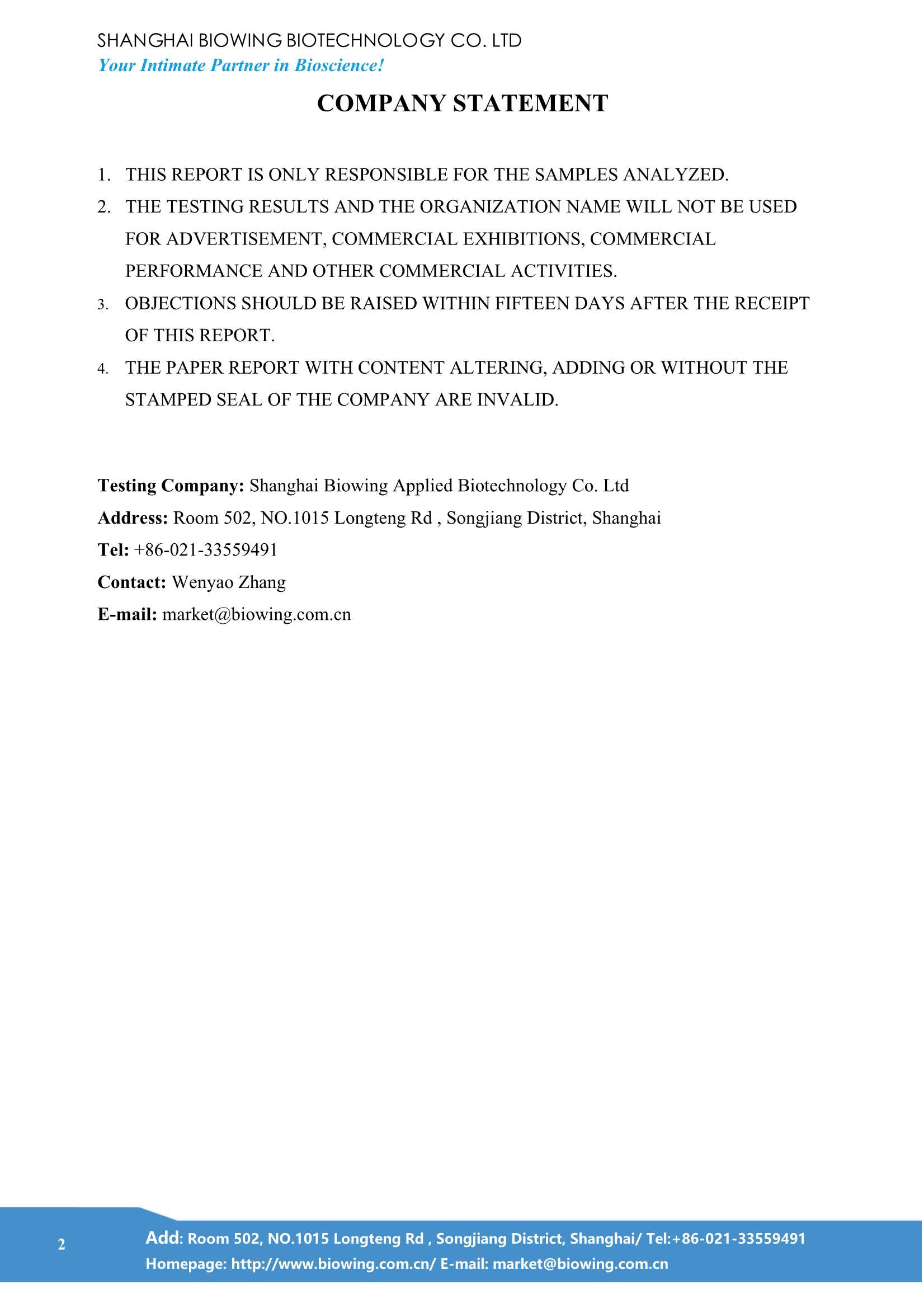

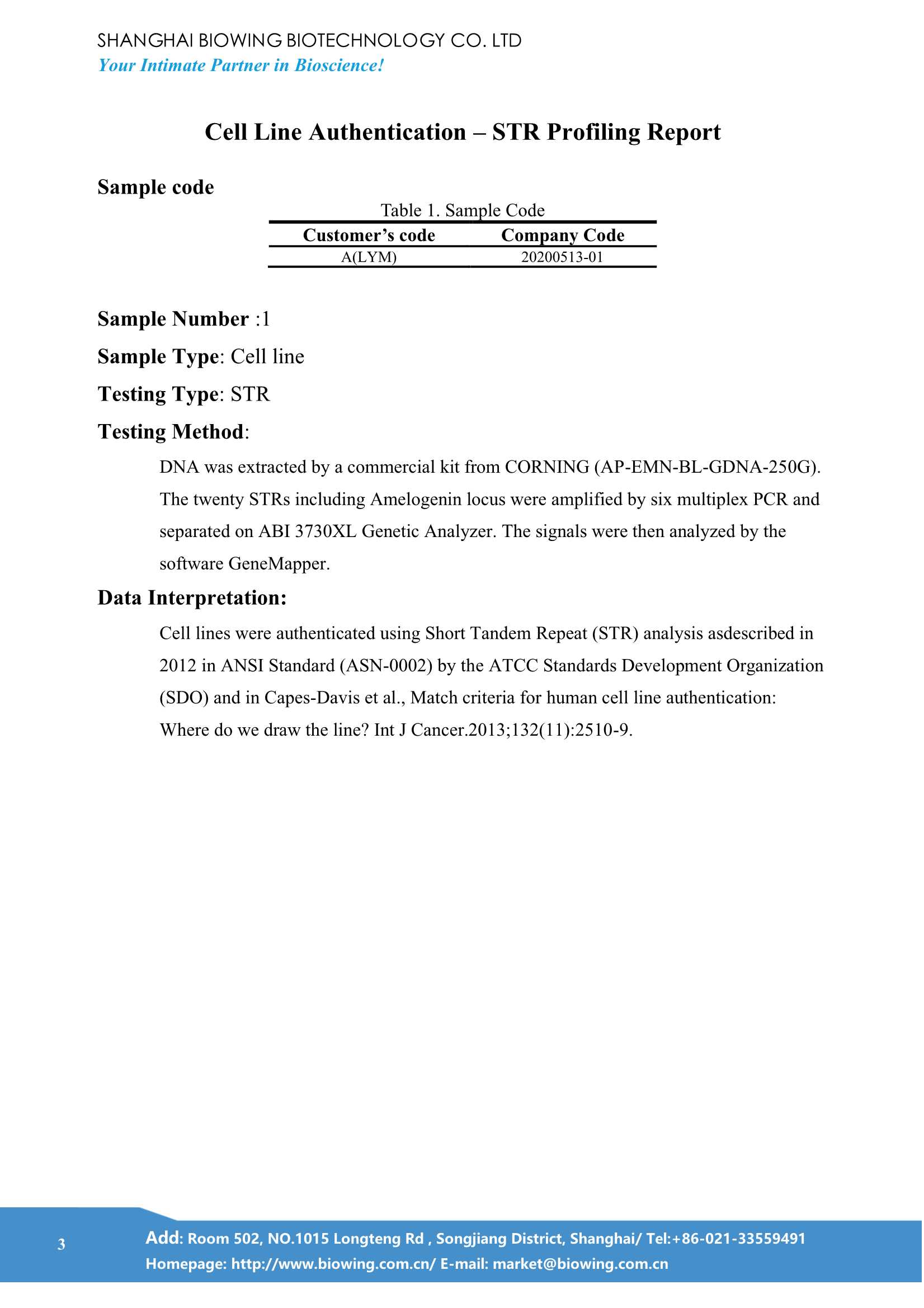

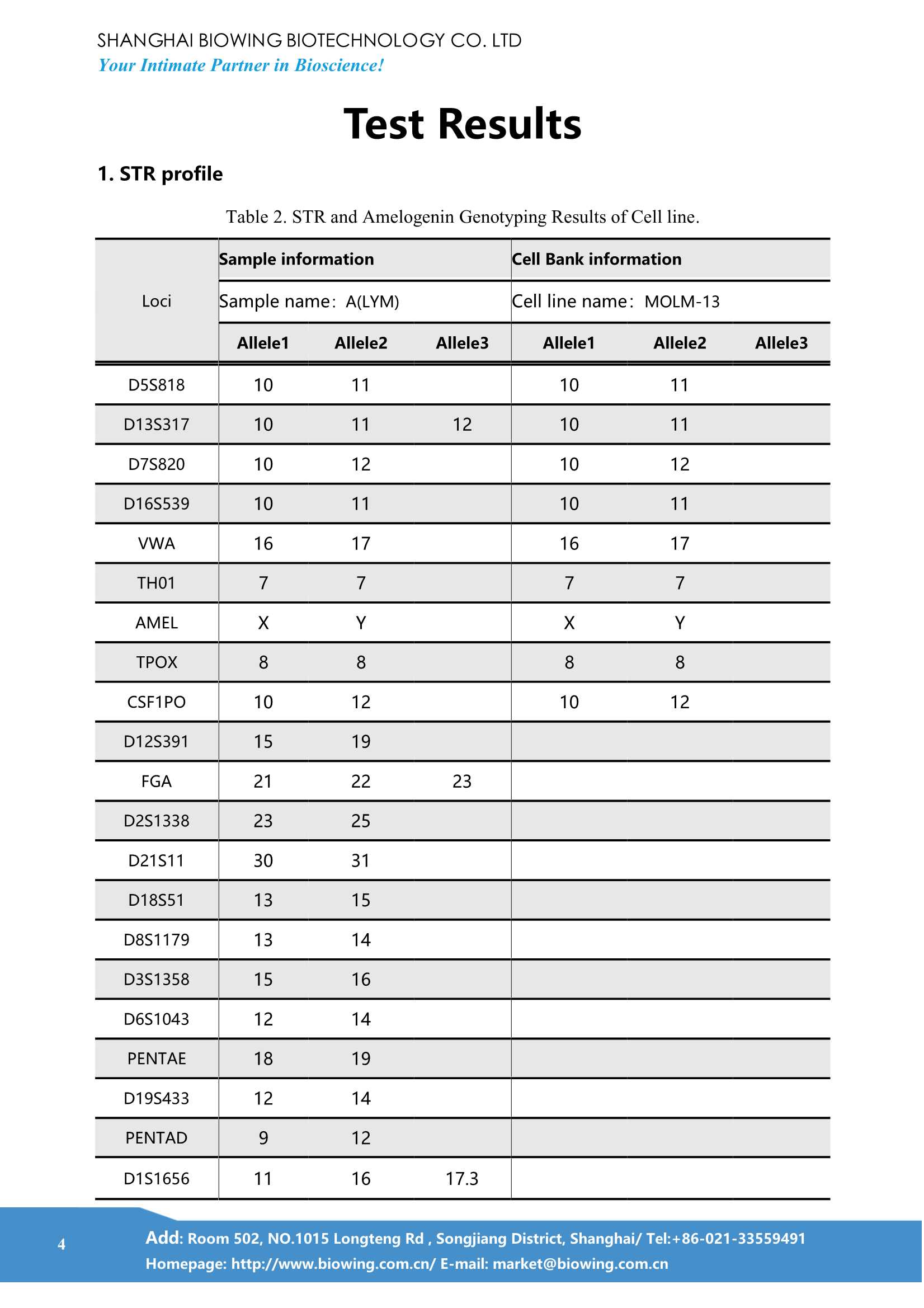

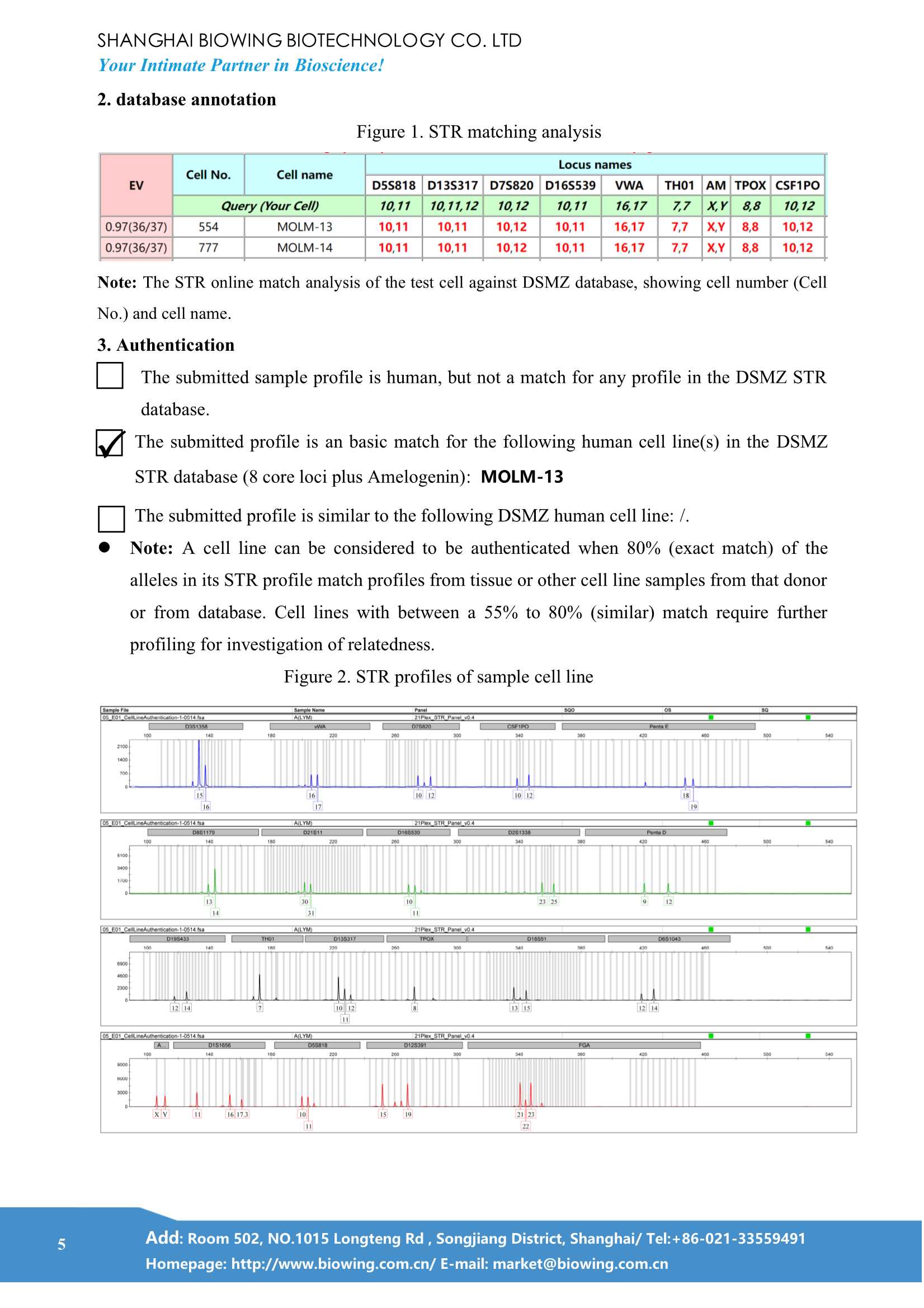

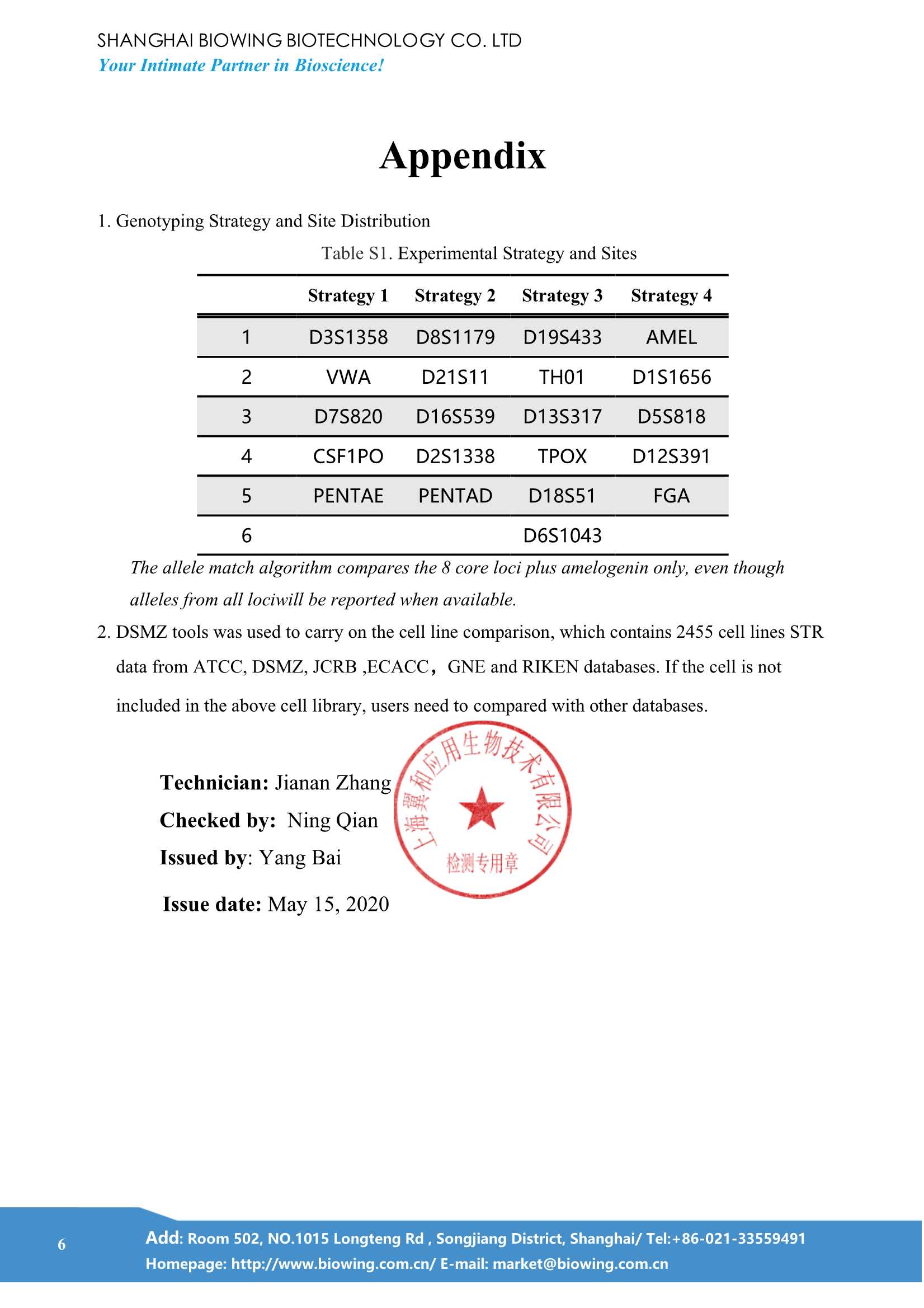


Supplementary Figure 1. MDS transformed AML ce­­ll line MOLM-13 was validated by short tandem repeat (STR) profiling.

**Supplementary Figure 2**


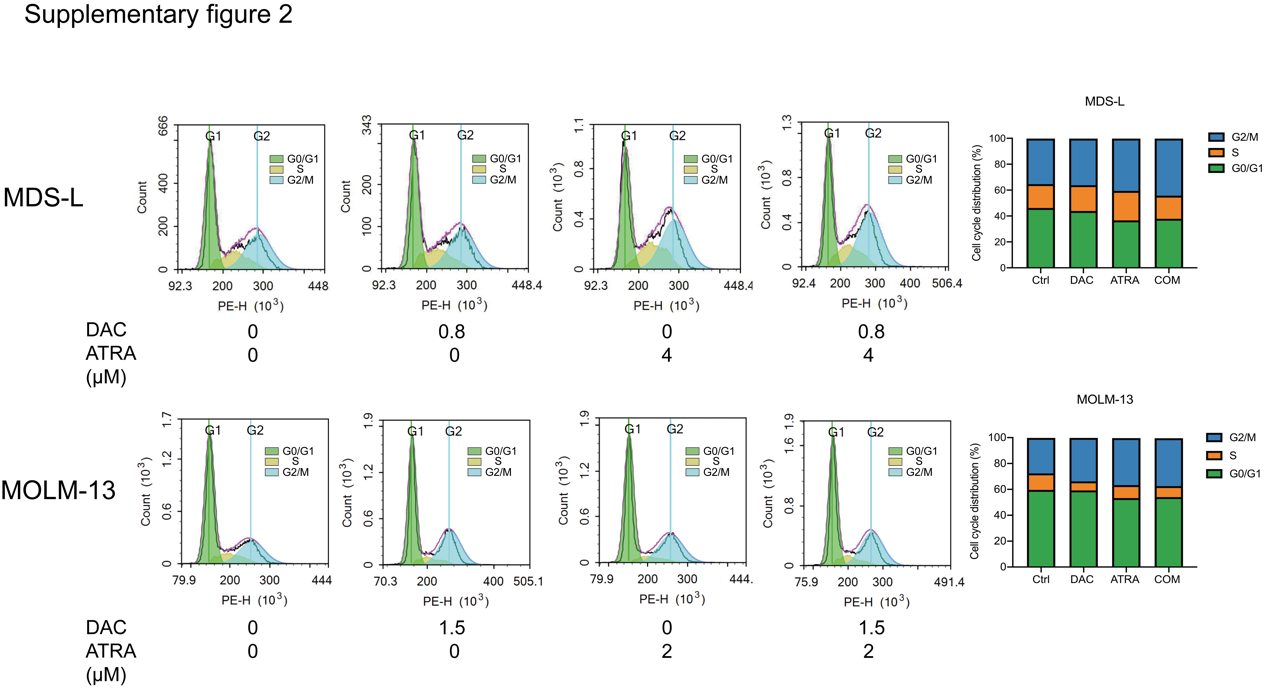


Supplementary Figure 2. After treatment with DAC and ATRA as a single agent or in combination, the cell cycle distribution of MDS-L and MOLM-13 cell lines had no significant difference. Abbreviations: DAC, decitabine; ATRA, all*-trans* retinoic acid.

**Supplementary Figure 3**


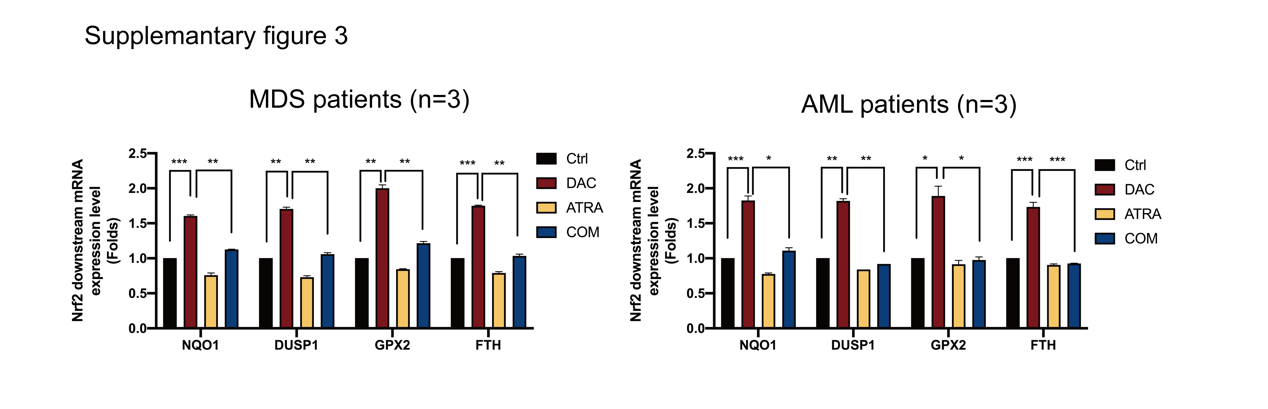


Supplementary Figure 3. Compared with Ctrl, treatment with DAC alone significantly increased the expression levels of NQO1, DUSP1, GPX2, and FTH of MDS and AML patients, while combination with DAC and ATRA markedly reversed the expression level of Nrf2 downstream genes. * *P* < 0.05, ** *P* < 0.01, *** *P* < 0.001.Abbreviations: Ctrl, control.

Supplementary Table 1. The *RARA* shRNA sequence.

| *RARA* sh1-F | GATCCGGTATTAATTCTCGCTGGTTTTCAAGAGAAACCAGCGAGAATTAATACCTTTTTTGG |
| --- | --- |
| *RARA* sh1-R | AATTCCAAAAAAGGTATTAATTCTCGCTGGTTTCTCTTGAAAACCAGCGAGAATTAATACCG |
| *RARA* sh2-F | GATCCCCCAAGATGCTAATGAAGATTTCAAGAGAATCTTCATTAGCATCTTGGGTTTTTTGG |
| *RARA* sh2-R | AATTCCAAAAAACCCAAGATGCTAATGAAGATTCTCTTGAAATCTTCATTAGCATCTTGGGG |

Abbreviations: *RARA*, retinoic acid receptor alpha.

Supplementary Table 2. Baseline characteristics of the MDS and AML patients enrolled for primary cellular viability research.

|  | Gender | Age | Diagnosis | Subtype | Karyotype | Genetic mutation | Fusion gene |
| --- | --- | --- | --- | --- | --- | --- | --- |
| MDS#1 | Male | 63 | MDS | EB1 | 46, XY [20] | *SF3B1, TET2, ASXL1* | None |
| MDS#2 | Male | 82 | MDS | EB2 | 46, XY [20] | NA | None |
| MDS#3 | Male | 63 | MDS | EB2 | 47, XY, +8[10] | *BCOR, IDH1, PHF6, RUNX1* | None |
| AML#1 | Female | 32 | AML | M5 | 47, XX, +22[2]  46, XX [1] | NA | *CBFB-MYH11* |
| AML#2 | Female | 42 | AML | M2a | 46, XX, t(8:21)(q22:q22)[2];  46, XX [8] | *NRAS, KIT, TET2, GATA2* | *AML1-ETO* |
| AML#3 | Female | 33 | AML | M2a | 46, XX [20] | *WT1, CEBPA* | None |
